# The impact of a daily pre-operative surgical huddle on interruptions, delays, and surgeon satisfaction in an orthopedic operating room: a prospective study

**DOI:** 10.1186/s13037-015-0057-6

**Published:** 2015-02-21

**Authors:** Avish L Jain, Kerwyn C Jones, Jodi Simon, Mary D Patterson

**Affiliations:** Children’s Hospital Medical Center of Akron, 590 Ridgecrest Rd, Akron, OH 44303 USA

**Keywords:** Daily, Pre-operative, Surgical, Huddle, Orthopedic, Interruptions, Satisfaction, Impact

## Abstract

**Background:**

The goal of this project was to implement a daily pre-operative huddle (briefing) for orthopedic cases and evaluate the impact of the daily huddle on surgeons’ perceptions of interruptions and operative delays.

**Methods:**

Baseline measurements on interruptions, delays, and questions were obtained. Then the daily pre-operative huddle was introduced. Surgeons completed a surgical outcomes worksheet for each day’s cases. Outcomes evaluated were primarily interruptions and delays starting cases before and following introduction of the huddle.

**Results:**

19 baseline observations and 19 huddle-implemented observations of surgeon’s days were assessed. Overall, surgeon satisfaction increased and fewer delays occurred after introduction of huddles. Interruptions decreased in all categories including equipment, antibiotics, planned procedure and side. Time required for a huddle was less than one minute per case.

**Conclusions:**

In this pilot study, a daily pre-operative huddle improved the flow of a surgeon’s day and satisfaction and indirectly provided indications of safety benefits by decreasing the number of interruptions and delays. Further studies in other surgical specialties should be conducted due to the promising results. Data was collected from three orthopedic surgeons in this phase; however, as a next step, data should be drawn from the rest of the orthopedic surgical team and other surgical subspecialties as well.

## Background

The World Health Organization (WHO) surgical checklist has been implemented worldwide and there is evidence that its use results in decreased morbidity and mortality for surgical patients [1]. However, this improvement in outcomes is heavily dependent on checklist compliance [2,3]. There is also evidence that compliance with the surgical checklist is highly variable and dependent on the ways in which the checklist is introduced, it’s perceived relevance and surgical team member engagement [4,5].

The WHO surgical checklist has not been embraced as widely in orthopedics as in some other surgical specialties [6-9]. It has been theorized the WHO surgical checklist is not as relevant to orthopedic procedures, and this has slowed its adoption among orthopedics and other surgical specialties.

Another concern that has been raised, related to the surgical checklist, is that it is often completed as the case is beginning. In some cases the checklist is completed in a “tick and flick” superficial approach [8] and in other cases the information surfaced during completion of the checklist occurs too late in the perioperative process to optimize care or avoid delays (i.e.: the patient is already anesthetized when it is recognized that antibiotics have not been administered or that necessary equipment is missing).

A complementary approach to the surgical checklist is the daily pre-operative huddle. The daily pre-operative huddle is an opportunity for the team to look at the entire day’s cases, identify potential problems, and set expectations prior to beginning the day’s cases. This helps to create a shared mental model for the entire team. Prior to starting this pilot study, one of the authors (KJ) tested a huddle, for a day with an unusually large caseload, to test the broader impact of huddles. The surgeon reported the day ran much more smoothly than usual and there were far fewer interruptions during the current cases to inquire about subsequent patient cases for that day. The daily huddle on that day required approximately seven minutes to complete in total. The daily pre-operative huddle is different from the surgical checklist timeout and complements but does not replace the checklist. Pre-surgical huddles have also been associated with improved safety climate in the operating room [10]. Clarifying questions related to the cases were never discouraged and are included in the Expected Safety Behaviors adopted by the organization. We describe the results of a pilot intervention to introduce surgical huddles in an orthopedic practice.

## Methods

The study was a quality improvement project that utilized the standard “Plan, Do, Study, Act” method. It was reviewed by the Institutional Review Board and determined not to require Institutional Review Board oversight. The study population consisted of surgical team members including employed orthopedic surgeons, anesthesiologists, nurse anesthetists, operating room nurses, operating room and anesthesia technicians, and residents. The study was carried out at two different campuses of a single institution.

**Figure 1:**
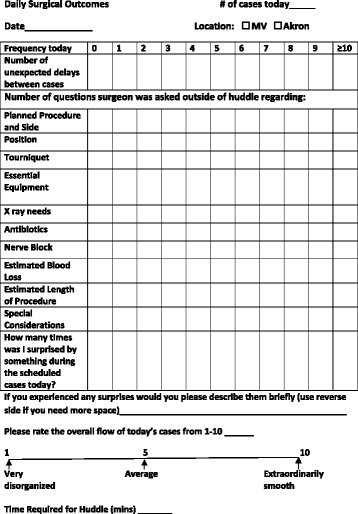
**Daily surgical outcomes.**

Over the course of one month, baseline observations of 19 surgeon days were recorded. Surgeons and their team proceeded through their day as usual. After the final case of the day, surgeons were asked to complete a questionnaire (Figure 1) about their day and record the number of questions they were asked from the surgical team regarding various categories, including equipment, antibiotics, and planned procedure. An interruption is defined as a question, related to the current case or another case, that the surgeon was asked during the course of an operation. For the purposes of this project, an interruption included questions that were otherwise addressed (or would have been addressed) during the huddle. Additionally, the surgeon noted the number of unexpected delays between cases, provided a rating on a scale of 1–10 of the flow of the day’s cases, and provided qualitative, both positive and negative, comments about the day. In most cases, the author (AJ) was present in the operating room and would document the interruptions in real time. (The observer directly recorded interruptions for 15 of the pre-intervention and 12 of the post-intervention surgeon days.) If questions occurred as to whether a particular incident constituted an interruption, the observer clarified the classification with the surgeon immediately following the case. If more than one surgeon was operating on the same day, the author would observe the surgeon performing the most cases, and the remaining surgeon would record and report the interruptions. When a surgeon was asked to record interruptions, he/she was provided the guidelines that each interruption outside of the huddle that is relevant to a case should be recorded. Interruptions that occurred while a procedure was taking place, in which the author was not present to record interruptions, were noted by the surgeon and recorded on the questionnaire.

**Figure 2:**
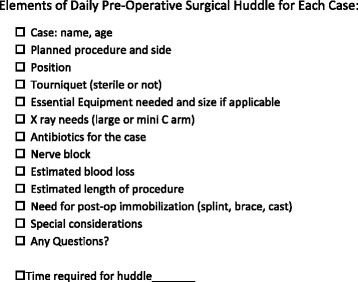
**Elements of daily pre-operative surgical huddle for each case.**

Following the baseline observations, the daily pre-operative huddle was implemented for six weeks which included a total of 19 surgeon days. The huddle was led by the attending surgeon in the presence of the entire surgical team and was completed prior to the first case of the day. At least one representative person was present at each huddle from anesthesia and surgical nursing, as well as the orthopedic surgeon leading the huddle. Surgeons were given a template (Figure 2) to follow for the huddle. The template was developed by the orthopedic team as a checklist of eleven critical elements that needed to be considered prior to the start of the procedure. The template was small enough to be attached to the surgeon’s identification badge. In addition, surgeons were instructed on the proper way to perform a huddle by author (KJ). All cases for the entire day were discussed, sequentially, at the beginning of the day with the surgical team. The elements on the template were discussed in detail for each planned case and discussion among the team was encouraged by the orthopedic surgeon asking the staff if there were any further questions or concerns at the end of the huddle. The surgical team progressed through the day as usual. Using the same baseline data collection technique, the surgeon completed the questionnaire (Figure 1) after the final case of the day, noting the same items that were assessed by the surgeon prior to the institution of huddles; however, the data collected after the institution of huddles also included the time required for the huddle.

The data collected was analyzed using run charts, graphs, and descriptive statistical analysis. In addition to observations, qualitative comments were solicited from members of the surgical teams as to how the daily preoperative huddle impacted their day.

The same three attending surgeons participated both at baseline and for the huddle intervention. Two of them are males and one is a female. Their ages ranged from 40–61 years old with the mean being 50 years old.

## Results

Table 1 shows the results from baseline observations and observations following huddle implementation. The mean time required for the entire huddle was 3.18 minutes or 0.98 minutes/case.

**Table 1:** **Comparison of results from baseline and huddle implementation observations**

	**Baseline (19 days, 65 cases)**	**Huddles (19 days, 62 cases)**
**Total Unexpected Delays**	15	4
**Rate of Unexpected Delay per** ***Case***	23.08%	6.45%
**Rate of Unexpected Delay per** ***Day***	78.95%	21.05%
**Total Questions Asked by All Surgical Teams** (outside of huddle)	163	35
**Questions per Case** (outside of huddle)	2.51	0.57
**Average Overall Flow Rating by Surgeons (1–10)**	5.58	8.16

**Figure 3:**
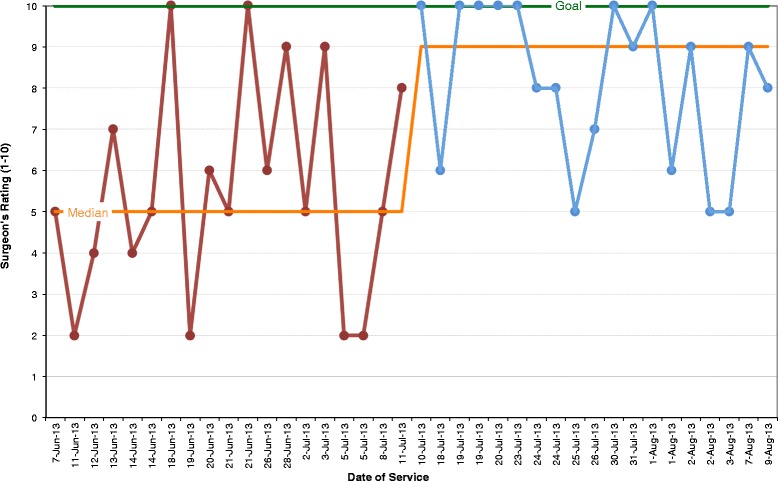
**Surgeon daily flow ratings before/with huddle implementation. Legend:**

**Figure 4:**
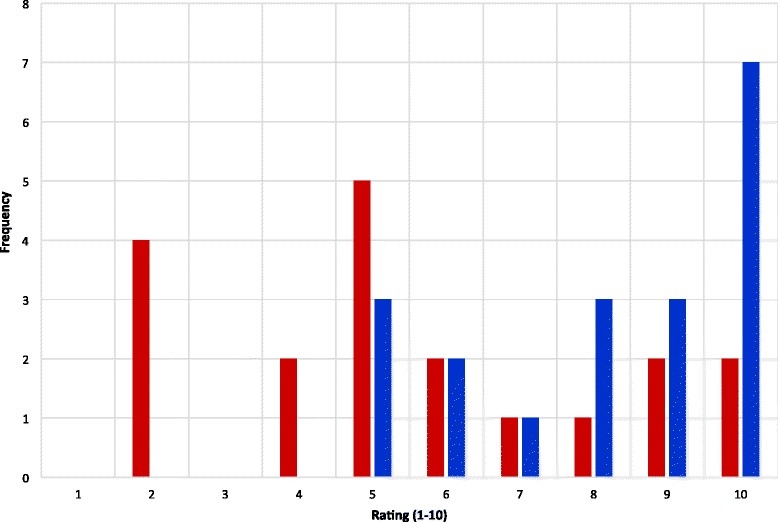
**Comparison of surgeon's flow of day rating before/with huddles. Legend:**

The huddles provided many significant advantages. Figure 3 shows the surgeons’ ratings at baseline and after huddle implementation for “day’s flow”. The median surgeon rating increased from a rating of 5 to 9 (10 being the best rating). Following huddle implementation, all ratings for surgeon perception of the cases were higher than the median rating at baseline. Figure 4 demonstrates the surgeon’s rating showing a comparison of the frequency of each number on the rating scale of 1–10. The highest rating of 10 (best rating) occurred almost 25% more often following huddle implementation. With a p-value less than 0.01, we can conclude that the average surgeon rating for the “day’s flow” after implementing huddles is higher. In addition, the mean difference of surgeon’s rating for the “day’s flow” before and after implementing huddles is between 1.3 and 3.9; thus, providing a rating between 6.9 and 9.4 before and after huddles.

**Figure 5:**
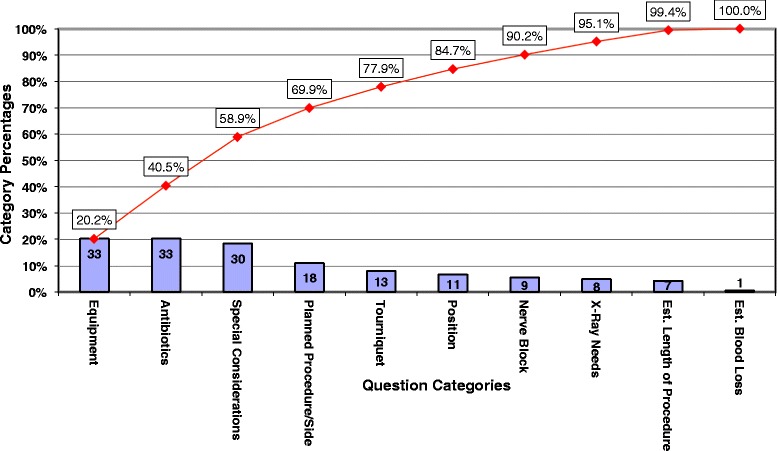
**Interruptions without surgical huddles. Legend:**

**Figure 6:**
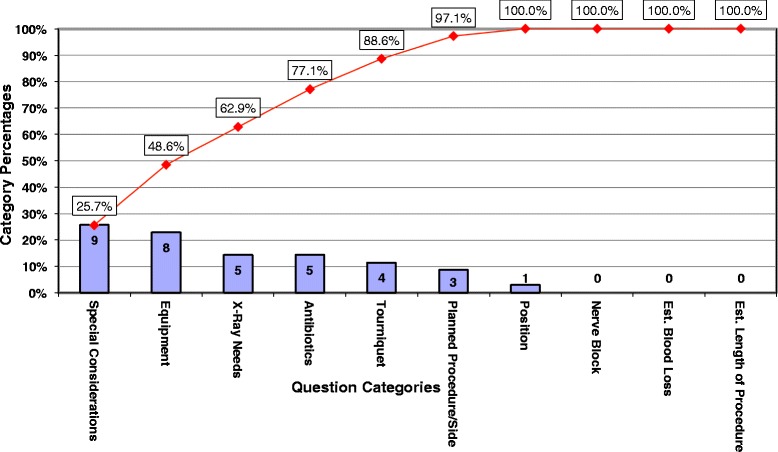
**Interruptions with surgical huddles. Legend:**

Figures 5 and 6 are Pareto charts representing the number of questions/interruptions during the surgeon’s day (outside of huddle) categorized by planned procedure and side, position, tourniquet, essential equipment, x-ray needs, antibiotics, nerve block, estimated blood loss, estimated length of procedure, and special considerations. Interruptions at baseline showed equipment and antibiotics as the most frequent categories, and planned procedure/side as the 4^th^ most frequent cause of interruption. Following huddle implementation, equipment dropped to the 2^nd^ most frequent cause of interruptions. Antibiotics and planned procedure dropped to the 4^th^ and 6^th^ most frequent cause of interruptions, respectively. The downward change in ranking of equipment interruptions has the potential to decrease delays and decrease instances of missing equipment for various procedures. The change in antibiotics may mean antibiotics are more likely to be given in the appropriate time frame thereby contributing to improving the surgical site infection rate. The change in planned procedure/side has the potential to minimize the cases where the wrong procedure is done or the wrong side is opened – an area where orthopedic surgeons are especially at high risk. Overall, with surgical huddles, the number of total interruptions decreased considerably (163 before to 35 after) and the number of questions per case (outside of huddle) was reduced by 77%.

## Discussion

The huddle before the start to the operating room day is a method to ensure that every team member has clarity regarding the surgical procedure and the patient identity? prior to the start of the day’s cases. The pre-operative huddle template (Figure 2) includes information about each planned case. Many of the elements discussed in the huddle are not included in the WHO surgical checklist. In addition, the surgeon’s portion of the surgical checklist is typically performed after the patient is anesthetized. We believe this is too late for identifying many of the critical needs that cannot be corrected at this point due to inadequate time to do so. One example of this is the need for specific equipment. Discussing special equipment is a routine part of the pre-operative huddle. Recognition of a missing device is much easier to correct and safer for the patient if it is identified prior to induction of anesthesia. If necessary, the order of surgical procedures during the day can be changed if this is recognized during a pre-operative huddle allowing time to obtain the critical equipment needed. We believed the different elements included in the “huddle” help the surgical team anticipate what equipment and resources will be needed as well as set expectations for the day’s cases. When expectations are established, deviations from the plan may be identified more easily.

The elements discussed in the huddle correlate to the elements assessed on the daily questionnaire. Therefore, the questionnaire was utilized as a method to determine if the huddle was directly affecting these specific areas of concern. The questionnaire addressed the number of questions related to surgical cases; many of which were preemptively addressed during the huddle such as the surgical position and the equipment needed to perform the operation. Therefore, interruptions while in the operating room were decreased. We observed that the huddle encouraged discussion among the surgical team by enabling the team to actively think about the cases and pose any questions or concerns prior to the start of the day’s cases. The goal of the huddle was to increase communication prior to the start of the day’s cases, clarify any issues that may cause problems later in the day for subsequent cases, and give ample time for any corrections.

Although it was not assessed in this study, it was felt that the huddle also increased the nursing staff satisfaction as evidenced by several nursing relevant notations in the “comments” section of the surgeons’ data sheets. In particular, immediately after a pre-operative surgical huddle one nurse remarked: “That was amazing, that was so helpful. I wish everyone would do this”. In this case, the nurse was a general surgery team member that was cross covering an orthopedic trauma case. The huddle may provide an even better understanding of expectations and the requirements for the surgical cases when the nurse, as in this case, is not typically involved in orthopedic procedures. Likewise, comments were encouraged from the surgeons involved in the trial. When one surgeon was asked how the huddles influenced their day, this surgeon said: “Helpful with big cases, unusual procedures, and those with a lot of instrumentation.” Another surgeon said: “Best way for 2-way communication under no duress . . . helps with non-elective cases and non-orthopedic nurses.”

The negligible time needed (about 54 seconds) to complete a huddle for each case was non-disruptive to workflow. The high ranking that surgeons gave the huddle suggests they view the minimal time investment as value added to the day.

Even though the number of questions outside of the huddle decreased, this was not the intent of the study. Instead, the data suggests questions were proactively asked and addressed in the huddle, thus, decreasing the need for questions during the operative cases. The huddle allows for an increase in time to adapt and develop solutions. During an operation, urgent situations may arise and the solutions used may not be optimal. The huddle prior to the start of the day allows for room to maneuver and establish optimal solutions for potentially difficult situations. As the use of huddles increase, we would expect the identification of optimal solutions would increase as the operating team discovered how to best utilize their time during the huddle. The huddle allows for not only improved efficiency of the OR team, but also makes the operative environment safer for everyone – surgeon, team, and patient. Specifically from our results, the implementation of the huddle may have the potential to reduce surgical site infections and wrong-side surgeries. However, taken broadly, the increase in communication among the surgical team members may reduce the potential for mistakes, accidents and unanticipated problems. A more-prepared team correlates to a safer environment.

As expected, a few problems were encountered during implementation. First, when OR staff changes for the next shift, typically in the mid-afternoon, proper handoffs are inconsistent due to competing demands. These include the requirement for employees to clock out on time. Second, it is possible the surgeon’s rating was a global rating, and that the rating was not specific to the elements addressed in the huddle. Lastly, at times it was difficult to sequester a representative member from anesthesia and surgical nursing due to the number of duties required of them prior to the start of the day. However, as the huddle process continued, culture evolved and it became easier to assemble the necessary individuals to expedite a proper pre-operative huddle. Clearly, the team found the nominal time required to perform the huddle to be value added to the day.

Albeit the study was a pilot study, there were indeed a few limitations. First, a few days of the surgeon interruptions were self-recorded rather than recorded by an observer. However, the mean number of interruptions per case was similar for the surgeon that self-recorded interruptions compared to those that were observed. Second, only three surgeons were involved and the study was done in a single institution in a pediatric orthopedic environment. This may not truly represent the impact a huddle may have on both the surgeon’s and surgical team’s satisfaction as well as the patient safety implications. However, it can be said that there seems to be a general benefit from all three of these perspectives that accompanies implementation of the huddle in this setting and that this deserves to be explored in other operative settings.

Some may foresee the time needed to conduct a huddle prior to the start of the day for high-volume departments with a large number of surgeries as prohibitive. It may also be suggested that much of the information shared during a huddle is available on the operative schedule or surgeon’s preference card. However despite these standard sources of information, at baseline the number of interruptions and delays due to missing equipment and/or questions concerning position or antibiotic administration were substantial in our setting. It is possible that the variation associated with pediatric practice and pediatric patients exaggerates the positive effect of a surgical huddle. In a more standard general orthopedic setting, many of the procedures may be the same or very similar to one another. It is important to understand the value of a huddle in a more general setting and also to evaluate the possibility of grouping discussion about similar cases to reduce the time a huddle would require. A pre-operative huddle is likely still a valuable practice in high-volume departments and the challenge is the best way to minimize the time required for a huddle without foregoing critical elements of the huddle that add value. Additionally, some of the information discussed in the huddle can be recorded on an OR schedule for each case to minimize the time needed in the huddle for usual items and allow time for discussion of the unusual elements.

Future studies are planned as a result of the promising qualitative and quantitative data from this pilot. We plan to recruit other surgical specialties to conduct huddles in order to determine if the positive results seen with orthopedic surgeries are replicated with other surgical specialties. Additionally, it is important to understand the impact of the huddle on non-surgeon members of the operating room team. A future study will also examine what particular types of checklist elements are necessary and useful with other types of cases. Concurrently, the study will look at what types of questions are being asked that are not currently being addressed by our huddle. Lastly, the ultimate goal is to understand if these types of results can be replicated across different organizations with varying cultures and processes.

## Conclusion

In this pilot, daily pre-operative huddles for orthopedic surgical procedures clearly improve the flow of a surgeon’s day and increases the surgeon’s satisfaction. The number of interruptions and delays during and between cases decreased. Lastly, the huddles may provide safety benefits associated with better preparation of the surgical team and a clearer shared mental model between the surgeon and the surgical team. There were logistic challenges associated with the implementation of the huddle, some of which were mitigated as the value of the huddle was embraced by members of the surgical team. Future studies should be conducted to test huddles with other surgical specialties and to assess the impact of the huddle on non-surgeon staff. Importantly, the benefits from a daily huddle are the result of taking only a few minutes prior to beginning the surgeon’s daily cases. Huddles have the potential to improve the functioning of the operating room team and to make the surgical experience better for the patient and the staff.
